# Random walks on mutual microRNA-target gene interaction network improve the prediction of disease-associated microRNAs

**DOI:** 10.1186/s12859-017-1924-1

**Published:** 2017-11-14

**Authors:** Duc-Hau Le, Lieven Verbeke, Le Hoang Son, Dinh-Toi Chu, Van-Huy Pham

**Affiliations:** 1 Vinmec Research Institute of Stem Cell and Gene Technology, 458 Minh Khai, Hai Ba Trung, Hanoi, Vietnam; 2 Department of Information Technology, Ghent University – imec, Ghent, Belgium; 3 VNU University of Science, Vietnam National University, Hanoi, Vietnam; 4 Faculty of Biology, Hanoi National University of Education, Hanoi, Vietnam; 5 Institute of Research and Development, Duy Tan University, 03 Quang Trung, Da Nang, Vietnam; 6 Faculty of Information Technology, Ton Duc Thang University, Ho Chi Minh City, Vietnam

**Keywords:** Disease-associated microRNAs, Network analysis, microRNA targets, Random walk with restart

## Abstract

**Background:**

MicroRNAs (miRNAs) have been shown to play an important role in pathological initiation, progression and maintenance. Because identification in the laboratory of disease-related miRNAs is not straightforward, numerous network-based methods have been developed to predict novel miRNAs in silico. Homogeneous networks (in which every node is a miRNA) based on the targets shared between miRNAs have been widely used to predict their role in disease phenotypes. Although such homogeneous networks can predict potential disease-associated miRNAs, they do not consider the roles of the target genes of the miRNAs. Here, we introduce a novel method based on a heterogeneous network that not only considers miRNAs but also the corresponding target genes in the network model.

**Results:**

Instead of constructing homogeneous miRNA networks, we built heterogeneous miRNA networks consisting of both miRNAs and their target genes, using databases of known miRNA-target gene interactions. In addition, as recent studies demonstrated reciprocal regulatory relations between miRNAs and their target genes, we considered these heterogeneous miRNA networks to be undirected, assuming mutual miRNA-target interactions. Next, we introduced a novel method (RWRMTN) operating on these mutual heterogeneous miRNA networks to rank candidate disease-related miRNAs using a random walk with restart (RWR) based algorithm. Using both known disease-associated miRNAs and their target genes as seed nodes, the method can identify additional miRNAs involved in the disease phenotype. Experiments indicated that RWRMTN outperformed two existing state-of-the-art methods: RWRMDA, a network-based method that also uses a RWR on homogeneous (rather than heterogeneous) miRNA networks, and RLSMDA, a machine learning-based method. Interestingly, we could relate this performance gain to the emergence of “disease modules” in the heterogeneous miRNA networks used as input for the algorithm. Moreover, we could demonstrate that RWRMTN is stable, performing well when using both experimentally validated and predicted miRNA-target gene interaction data for network construction. Finally, using RWRMTN, we identified 76 novel miRNAs associated with 23 disease phenotypes which were present in a recent database of known disease-miRNA associations.

**Conclusions:**

Summarizing, using random walks on mutual miRNA-target networks improves the prediction of novel disease-associated miRNAs because of the existence of “disease modules” in these networks.

**Electronic supplementary material:**

The online version of this article (10.1186/s12859-017-1924-1) contains supplementary material, which is available to authorized users.

## Background

MiRNAs are a class of small non-coding regulatory RNAs that play an important role in the regulation of gene expression [1, 2]. Misregulation of miRNAs has been shown to contribute to both common [3–7] and rare diseases [8]. Because the identification in the laboratory of miRNAs related to a particular disease is non-trivial, computational methods for the in silico identification of potential disease-miRNAs associations have great potential for speeding up this process.

A number of computational methods, mostly network-based or machine learning approaches, have been proposed for the prediction of disease-associated miRNAs [9]. The network-based methods mainly rely on the construction of similarity networks expressing functional similarities between miRNAs, after which specific algorithms are used to detect novel disease-miRNA associations [10–20]. Recently, disease similarity matrices have been additionally integrated with the miRNA functional similarity network to construct heterogeneous networks of diseases and miRNAs, using known disease-miRNA associations [21–25].

Most often, the similarity networks used are functional miRNA similarity networks, containing only miRNAs as nodes (hereafter referred to as homogeneous miRNA networks). In these networks, nodes represent miRNAs and edges represent the degree of functional relatedness between the miRNAs. This functional relatedness can be derived from miRNA-target gene interactions in different ways. For example, miRNA functional similarity interactions were constructed based on the degree to which miRNAs share the same targets [10] or by calculating the similarity of target gene regulation patterns for each pair of miRNAs [11]. Additionally, Wang et al. [12] assessed the functional similarity between two miRNAs by comparing the gene functions (using gene ontologies) of their respective sets of target genes. Similarly, Xu et al. [13] constructed functional synergistic regulatory interactions between miRNAs by considering common target genes in the context of gene ontology and proximity in a protein interaction network. All these methods capture a different aspect of functional similarity, and we demonstrated previously that there can be added value in constructing a functional similarity network by integrating functional similarity interactions obtained using several of the aforementioned methods [14].

Once a homogeneous miRNA networks is available, associations between miRNAs and diseases are subsequently predicted by assuming that functionally related miRNAs associate with phenotypically similar diseases, which is referred to as the “disease module” principle [26, 27]. Specific methods that exploit this principle have been proposed. Local similarity measures only assess direct neighbours of known disease-associated miRNAs [10, 11] or neighbours of candidate miRNAs (as used e.g. by HDMP [17]) in homogeneous miRNA networks. Another state-of-the-art method for disease miRNA prediction, RWRMDA [14, 15], obtains a global network similarity metric by running a random walk with restart (RWR) algorithm (a network propagation technique) on homogeneous miRNA networks. RWR-based techniques were also applied on different network types where either a phenotype similarity network [20] or a protein interaction network [28] was used as input for the analysis. In addition, we recently demonstrated that network-based ranking algorithms, which were successfully applied for either disease gene prediction or for studying social networks and networks of interlinking web pages, could also be used effectively for disease microRNA prediction on homogeneous miRNA networks, achieving comparable performance with the RWR-based method [16]. For heterogeneous networks of diseases and miRNAs, pathfinding-based methods were used [21, 22] that rely on the assumption that the more paths exist between a miRNA and a disease, the more likely it is that there exists an association between them. In addition, based on the assumption that functionally similar miRNAs tend to be associated with similar diseases, other methods were proposed relying on the identification of clusters of similar diseases and similar miRNAs [23–25].

Next to network-based methods, machine learning-based methods that do not use miRNA-target interactions have also been proposed. For example, a Naïve Bayes model was used to integrate genomic data for prioritizing disease-related miRNAs [29]. Qinghua et al. [30] applied support vector machines for identifying disease-associated miRNAs. In addition, Qabaja et al. [31] used a Lasso regression model to infer disease-miRNA associations. The common limitation of these machine learning methods is the necessity to compile a set of negative training samples consisting of non-disease-related miRNAs. As the absence of an observed association does not imply the non-existence of an association (there are no proven negatives), obtaining such a negative training set is not straightforward [32]. More recently, RLSMDA [33], a semi-supervised classifier-based method, was proposed to overcome this limitation, prioritizing candidate miRNAs for all considered diseases without the need for negative samples. Importantly, RLSMDA was reported to outperform the aforementioned state-of-the-art methods RWRMDA [15] and HDMP [17].

A common limitation of the homogeneous miRNA network-based methods is that the knowledge of biological relationship between miRNAs and their target genes might be used ineffectively because this relationship is only partially integrated in the metric used to capture degree of similarity between two miRNAs. Also, the application of the RWR algorithm, underpinning several state-of-the-art network-based algorithms, is not limited to homogeneous networks containing only miRNA nodes. It can be applied to heterogeneous networks where both miRNAs and their gene targets are present in the network as nodes, and edges represent miRNA-target interactions. With the human genome containing thousands of miRNAs [34, 35], regulating the expression of thousands of genes [36, 37] and with these miRNA-target interactions (predicted or experimentally validated) now being largely available in a number of miRNA-target databases (as comprehensively reviewed in [38]), here we propose to use heterogeneous networks as input for the identification of disease-related miRNAs, in order to make optimal use of this increased level of detail.

MiRNAs have emerged as key regulators of gene expression in diverse biological pathways; the relationship of a miRNA and its target genes are usually considered as direct interactions between the miRNA and the target genes (i.e., a miRNA regulates target genes by binding to target sequences in mRNAs). Consequently, miRNA-target gene regulatory interactions were used as directed interactions in a number of studies [32, 39, 40]. However, recent developments introduced a new twist to this: targets can reciprocally control the level and function of miRNAs [41]. This mutual regulation of miRNAs and target genes in combination with the large coverage of miRNA-target interactions available in publicly available miRNA-target databases [38] has inspired us to propose a novel network-based method for disease miRNA prediction. In this study, instead of constructing homogeneous miRNA networks from target genes or using directed miRNA-target gene interactions, we exploit the mutual regulatory relations between miRNAs and their target genes to construct mutual heterogeneous miRNA-target gene networks (hereafter, referred to as mutual heterogeneous miRNA networks). Next, we propose a novel framework, RWRMTN, in which we apply the RWR algorithm on these heterogeneous miRNA networks to prioritize candidate disease miRNAs. In particular, based on a previous study indicating that miRNAs regulate diseases through their target genes [28], we hypothesize that the mutual regulation between a miRNA and their targets leads to a transfer of disease information between them. Therefore, in the proposed method, we force the RWR algorithm to start from a set of seed nodes, consisting not only of known disease miRNAs but also of their target genes. To assess and evaluate the predictive performance of RWRMTN, we use a leave-one-out cross-validation scheme on a set of experimentally verified disease phenotype-miRNA associations. Experimental results indicate that RWRMTN outperforms RWRMDA [15], a state-of-the-art network-based method using RWR operating on homogeneous miRNA networks. Additionally, we demonstrate that this superior performance of our proposed method is because of the existence of “disease modules” in the heterogeneous miRNA networks used as input for our algorithm. Indeed, we observe that (1) a large amount of known disease genes are present in the heterogeneous miRNA networks and (2) most known disease miRNAs in the network regulate at least one known disease gene. Moreover, we showed that our method also outperformed RLSMDA [33], a state-of-the-art machine learning-based method that uses a semi-supervised learning method. Furthermore, we demonstrated that our method is stable and can achieve relative high performance for both experimentally validated and predicted miRNA-target gene interaction data. Finally, using RWRMTN, we identified 76 novel miRNAs associated with 23 disease phenotypes which were present in an recent database of known disease-miRNA associations HMDD [42].

## Methods

### Construction of heterogeneous miRNA networks

**Fig. 1:**
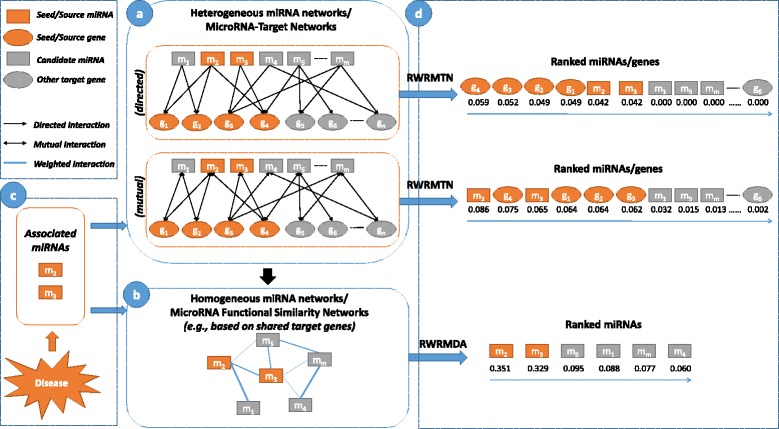
Illustration of the RWRMTN and RWRMDA methods. **a** Heterogeneous miRNA networks/MiRNA-target networks were constructed using miRNA-target gene interactions. **b** Homogeneous miRNA networks/MiRNA functional similarity networks were constructed using target genes shared among miRNAs. **c** Two miRNAs known to be associated with a disease under study are mapped as source/seed nodes in a homogeneous miRNA network. In addition to these two known disease-associated miRNAs, their target genes are also used as source/seed nodes in a heterogeneous miRNA network. **d** Ranking methods score all nodes in the heterogeneous or homogeneous miRNA network

To construct heterogeneous miRNA networks, we selected miRWalk [43], a database of experimentally validated miRNA-target interactions and TargetScan [44], a database containing predicted interactions. More specifically, we downloaded experimentally validated human miRNAs-target interactions from the miRWalk database and constructed a heterogeneous miRNA network consisting of 12,721 nodes (745 miRNAs and 11,976 genes) and 38,571 interactions (from now on referred to as *HetermiRWalkNet*) (See in Additional file 1: Table S1). This network can be considered as either a mutual heterogeneous miRNA network (*HetermiRWalkNet-mutual*) if the interactions between miRNAs and target genes are considered to be reciprocal, or alternatively as a directed heterogeneous miRNA network (*HetermiRWalkNet-directed*) if miRNAs are assumed to regulate target genes but not vice versa. In addition, we downloaded predicted human miRNA-target gene associations from TargetScan with non-conserved site context++ scores, and constructed a second heterogeneous miRNA network consisting of 16,568 nodes (1547 miRNAs and 15,021 genes) and 520,526 interactions (*HeterTargetScanNet*) (See in Additional file 1: Table S2). Again, this network can be considered as either a mutual heterogeneous miRNA network (*HeterTargetScanNet-mutual*) or a directed heterogeneous miRNA network (*HeterTargetScanNet-directed*). Figure 1a gives an overview of the different types of miRNA networks used in this study.

### Construction of homogeneous miRNA networks

To compare the prediction performance of RWRMTN with that of RWRMDA [15] on homogeneous miRNA networks, we constructed two homogeneous miRNA networks based on miRNA-target gene interactions (Fig. 1b). More specifically, based on an identical procedure of construction of homogeneous miRNA network as in our previous study [16], we defined a functional relation between two miRNAs as follows: two miRNAs are considered to be functionally interacting if they share at least one target gene, with the degree of similarity defined as the number of shared target genes normalized by the minimum number of target genes of the two miRNAs under consideration. As a result, two networks respectively containing 730 miRNAs with 29,089 interactions (*HomomiRWalkNet*) and 1428 miRNAs with 46,118 interactions (*HomoTargetScanNet*) are constructed from the miRNA-target gene interactions in *HetermiRWalkNet* and *HeterTargetScanNet*.

### Database of known disease phenotype-miRNA associations

In order to be able to evaluate the performance of the propose method, and to put the new method in perspective, a database of known disease-miRNA associations is required. Here we will use miR2Disease [45], a comprehensive resource of miRNA - human disease associations that is manually curated and maintained. We used 270 manually curated disease phenotype–miRNAs associations between 53 disease phenotypes and 118 miRNAs from that database (See in Additional file 1: Table S3).

### Construction of a disease phenotype similarity matrix

To compare the performance of RWRMTN and RLSMDA, we additionally collected a disease phenotype similarity matrix of 5080 phenotypes from [46], where an element of the matrix represents degree of similarity between two disease phenotypes. The similarities in this matrix were obtained by applying various text mining algorithms to OMIM records [47].

### RWRMTN: A random walk with restart algorithm applied to heterogeneous miRNA networks

RWR is a variant of the random walk algorithm, simulating a walker that either moves from a current node in a network to a randomly selected adjacent node or alternatively returns to the source node (also called the seed node) where the random walk was started, with a fixed probability of returning (restart probability) *γ*. This algorithm has been used successfully in a number of related studies such as prediction of disease-associated lncRNA [48], disease-associated gene [49], drug target [50] and disease-related microRNA-environmental factor interactions [51].

Given a connected weighted graph G(*V*, *E*) with a set of nodes *V* = {*v*_*1*_ *, v*_*2*_ *, …, v*_*N*_} and a set of links *E* = {(*v*_*i*_ *, v*_*j*_)| *v*_*i*_ *, v*_*j*_∈*V*}, a set of seed nodes *S*⊆*V*, and a *N*×*N* adjacency matrix *W*, the random walk with restart (RWR) can be formally described as follows:

1 $$ {p}_{t+1}=\left(1-\gamma \right){W}^{\hbox{'}}{p}_t+\gamma {p}_0 $$

Where *W′* represents a transition probability matrix and *W’*_*ij*_, the element in *W′* on row *i* and column *j*, denotes the probability that a random walker at node *v*_*i*_ moves to neighboring node *v*_*j*_:

2 $$ W{\hbox{'}}_{ij}=\frac{W_{ij}}{\sum_{k\in {\left({V}_{out}\right)}_i}{W}_{ik}} $$

Here

- (*V*_*out*_)_*i*_ is a set of outgoing nodes of *v*_*i*_. If an unweighted graph (e.g., a heterogeneous miRNA network) is used, all interactions are assigned a unity weight.
- *p*_t_ is a *N*×*1* probability vector of |*V*| nodes at a time step *t* of which the *i* ^*th*^ element represents the probability of the walker being at node *v*_*i*_∈*V*.
- *p*_*0*_ is the *N*×*1* initial probability vector.

In the RWRMDA method, the RWR technique is used to rank miRNAs in homogeneous miRNA networks. Therefore, the set of seed nodes *S* only contains known disease miRNAs (i.e., *S* = *S*_*m*_) and *p*_0_ is defined as follows:

3 $$ {\left({p}_0\right)}_i=\left\{\begin{array}{c}\frac{1}{\left|{S}_m\right|}\kern2em {ifv}_i\in {S}_m\\ {}\ 0\kern2.75em otherwise\end{array}\right. $$

Alternatively, for RWRMTN we assume that the mutual regulation between a miRNA and their targets leads to an exchange of disease information between the two entities participating in the interaction. Therefore, we enlarge the set of seed node *S* by adding target genes *S*_*g*_ of the known disease miRNAs (i.e., *S* = *S*_*m*_∪*S*_*g*_). The initial probability vector *p*_*0*_ is defined as follows:

4 $$ {\left({p}_0\right)}_i=\left\{\begin{array}{c}\alpha \frac{1}{\left|{S}_m\right|}\kern5em {ifv}_i\in {S}_m\\ {}\left(1-\alpha \right)\frac{1}{\left|{S}_g\right|}\kern2.5em {ifv}_i\in {S}_g\\ {}0\kern6.75em otherwise\end{array}\right. $$

where *α*∈[0, 1] is a weight parameter, controlling the amount of disease information transferred between miRNAs and their target genes.

For both methods, all miRNAs/genes in the network are eventually ranked according to the steady-state probability vector *p*_*∞*_, which is obtained by repeating the iterations until convergence is reached (in this study, ||*p*_t + 1_-*p*_t_|| <10^−6^).

Note that, for directed heterogeneous miRNA networks such as *HetermiRWalkNet-directed* and *HeterTargetScanNet-directed*, the random walker is trapped at seed target genes because there is no outgoing link at these nodes. Therefore, non-seed nodes (including previously unidentified disease miRNAs and other target genes) cannot be ranked as they are all assigned a zero probability (Fig. 1d). Therefore, RWRMTN can only be applied to mutual heterogeneous miRNA networks such as *HetermiRWalkNet-mutual* and *HeterTargetScanNet-mutual*. Figure 1 illustrates these two methods.

### RLSMDA: Regularized least squares for MiRNA-disease association

RLSMDA is a semi-supervised and global method since it can rank disease-miRNA associations for all diseases under consideration simultaneously, without the need for a negative training set. RLSMDA constructs a continuous function that can determine the association probability between each miRNA and a given disease. The higher this probability is, the more a miRNA is related to a given disease. To this end, RLSMDA relies on the minimization of two cost functions, defined in respectively the miRNA space and in the disease space, whose solutions are subsequently combined in a single continuous classification function [33]. The optimal classifier in these two spaces was defined as follows:

5 $$ {F}^{\ast }={wF}_M^{\ast T}+\left(1-w\right){F}_D^{\ast } $$

where ${F}_M^{\ast }$ and ${F}_D^{\ast }$ are optimal classification functions in the miRNA and disease phenotype spaces, respectively defined as:

6 $$ {F}_M^{\ast }={S}_M\left({S}_M+{\eta}_M{I}_M\right){A}^T $$

7 $$ {F}_D^{\ast }={S}_D\left({S}_D+{\eta}_D{I}_D\right)A $$

with

- *w* is the weight between these two spaces. *η*_*M*_ and *η*_*D*_ are trade-off parameters in the miRNA and disease phenotype spaces, respectively.
- *S*_*D*_(*m* × *m*) is the disease phenotype similarity matrix containing *m* diseases. *S*_*M*_(*n* × *n*) is the corresponding similarity matrix of the homogeneous miRNA network containing *n* miRNAs, where *S*_*M*_(*i*, *j*) is the degree of similarity between two miRNAs.
- *I*_*M*_ and *I*_*D*_ are identity matrices with the same size as matrices *S*_*M*_ and *S*_*D*_, respectively.
- *A*(*m* × *n*) is an association matrix, where *A* (*i*,*j*) = 1 if disease phenotype *i* is known to be associated with miRNA *j*, otherwise *A* (*i*,*j*) = 0.

### Performance evaluation

To compare the potential of RWRMTN for associating novel miRNAs with disease phenotypes with that of RWRMDA and RLSMDA, we applied a leave-one-out cross-validation (LOOCV) scheme on the set of disease phenotypes with known miRNA associations in miR2Disease [45]. For each disease phenotype *d*, in each round of LOOCV, we held out one known miRNA associated with *d*. The rest of the known miRNAs associated with disease *d* are used as seed nodes (*S*_*m*_) in the RWRMDA method. For the RWRMTN method, this set was enlarged by adding the target genes *S*_*g*_ of the miRNAs in *S*_*m*_. The held-out miRNA and the remaining miRNAs in the miRNA networks which were not known to be associated with *d*, were ranked by both RWRMTN and RWRMDA. For RLSMDA, *A* (*i*,*j*) is set to 0 corresponding to *d* and the held-out miRNA. Then, receiver operating characteristic (ROC) curves are constructed and the area under the curve (AUC) is used to compare the performance of both methods. The ROC curve represents the relationship between *sensitivity* and (*1-specificity*), where *sensitivity* refers to the percentage of miRNAs known to be associated with *d* that were ranked above a particular threshold and *specificity* refers to the percentage of miRNAs that were not known to be associated with *d* and ranked below this threshold. Finally, the performance of each method was summarized as the average of AUC values over the entire set of disease phenotypes in the validation set.

## Results and discussion

### Parameter settings

**Fig. 2:**
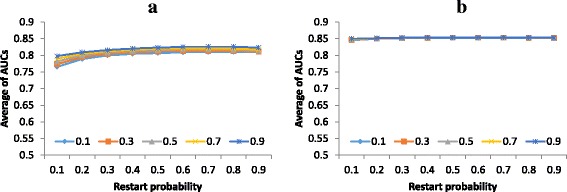
Performance of RWRMTN as a function of the algorithm parameters, using mutual heterogeneous miRNA networks. Performance is an average of AUC values over a set of disease phenotypes collected from the miR2Disease database [45]. The restart probability *γ* was varied in the range [0.1, 0.9]. The weight parameter α) was set to values in {0.1, 0.3, 0.5, 0.7, 0.9}. Results are reported for (**a**) *HetermiRWalkNet-mutual* and (**b**) *HeterTargetScanNet-mutual*

To determine the best setting for RWRMTN, we varied the weight parameter (α) in the range {0.1, 0.3, 0.5, 0.7, 0.9} and the restart probability *γ* in the range [0.1, 0.9] in steps of 0.1. For each combination of parameter values, we only assessed the performance of RWRMTN on mutual heterogeneous miRNA networks as the method cannot be applied to directed heterogeneous miRNA networks (See Materials and Methods). Performance was assessed as the average AUC over the set of disease phenotypes in the disease phenotype set (See Materials and Methods). Fig. 2a and b shows that the performance of RWRMTN slightly increased according to the change of the weight parameter on mutual heterogeneous miRNA networks constructed from miRWalk (*HetermiRWalkNet-mutual*) and from TargetScan (*HeterTargetScanNet-mutual*). This indicates that disease information contained in known disease miRNAs is still more important than that in their target genes when prioritizing candidate disease-associated miRNAs. In addition, optimal performance was achieved for both networks with α = 0.9 and γ = 0.7. For the RLSMDA method, we used the parameter settings (*η*_*M*_ = *η*_*D*_ = 1,  *w* = 0.9) reported in the corresponding study [33].

### Performance comparison

In this section, we compare the performance of RWRMTN with two state-of-the-art methods. We selected RWRMDA [15] as a representative network-based method, as we intended to demonstrate the added value of using heterogeneous miRNA networks over using homogeneous miRNA networks. Additionally we compared with RLSMDA [33], a state-of-the-art machine learning-based method, that does not use a network as a basis for its analysis.

#### Comparison between RWRMTN and RWRMDA

**Fig. 3:**
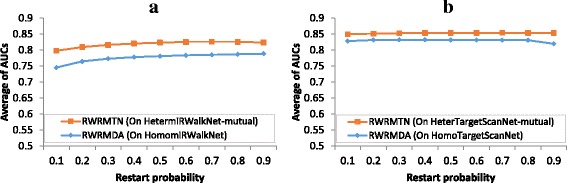
Performance comparison between RWRMTN and RWRMDA. The performance of each method on each heterogeneous/homogeneous miRNA network is calculated as the average AUC values over a set of disease phenotypes collected from the miR2Disease database [45]. The restart probability was varied from 0.1 to 0.9. The weight parameter was set to 0.1. **a** Comparison between RWRMTN (using *HetermiRWalkNet-mutual)* and RWRMDA (using *HomomiRWalkNet).* **b** Comparison between RWRMTN (using *HeterTargetScanNet-mutual)* and RWRMDA (using *HomoTargetScanNet)*

In a previous study [16], we demonstrated that other homogeneous miRNA network-based methods achieve performance similar to RWRMDA [15], a RWR-based method. Therefore, in this study, we only compare the prediction performance of RWRMTN on the heterogeneous miRNA networks with that of RWRMDA on the homogeneous miRNA networks. More specifically, we tested the performance of RWRMTN on the two mutual heterogeneous miRNA networks, *HetermiRWalkNet-mutual* and *HeterTargetScanNet-mutual,* and the performance of RWRMDA on the two homogeneous miRNA networks, *HomomiRWalkNet* and *HomoTargetScanNet.* In all experiments, we varied the random walker’s restart probability *γ* in a range of [0.1, 0.9] for both methods, and set the weight parameter α of RWRMTN to 0.9. The performance of both methods on each heterogeneous/homogeneous miRNA network is expressed as the average AUC values over the set of available disease phenotypes. Figure 3 shows the prediction performance of the two methods on heterogeneous/homogeneous miRNA networks constructed from miRWalk and TargetScan databases respectively. Analyzing the performance of the two methods on different heterogeneous/homogeneous miRNAs networks, we observed that the performance of RWRMDA on *HomomiRWalkNet* and *HomoTargetScanNet* was respectively slightly better and stable when the restart probability *γ* increased (the slopes of regression line are respectively 0.045 and −0.006 with *p* = 0.001 and *p* = 0.239, Fig. 3). This difference in performance response to the restart probability (increase vs. stable) when using different networks as input can be explained by the fact that when the restart probability is small, the random walker is able to travel relatively far from the seed nodes. This in turn allows for an improved exploitation of the “disease module” principle since it tends to assign higher scores to nodes close to the seed nodes. Therefore, the stable performance of RWRMDA as a function of the restart probability on homogeneous miRNA networks suggests that disease miRNAs are relatively close or directly connected to each other in the individual homogeneous miRNA networks. The increase in performance (when varying *γ*) observed when using *HomomiRWalkNet* suggests that disease miRNAs in this network are less modularized than those in *HomoTargetScanNet.*

In contrast to the homogeneous miRNA networks, miRNAs connect to each other via target genes in the heterogeneous miRNA networks. In other words, disease miRNAs are less modularized in these networks. Indeed, Fig. 3 show that the performance of RWRMTN slightly increased when the restart probability increased in both networks (the slopes of regression lines are 0.029 and 0.004 with *p* = 0.004 and *p* = 0.011, respectively for *HetermiRWalkNet-mutual* and *HeterTargetScanNet-mutual)*. It is also slightly more positive on *HetermiRWalkNet-mutual* indicating that disease miRNAs/genes in that network is less modularized than those in *HeterTargetScanNet-mutual.*

**Fig. 4:**
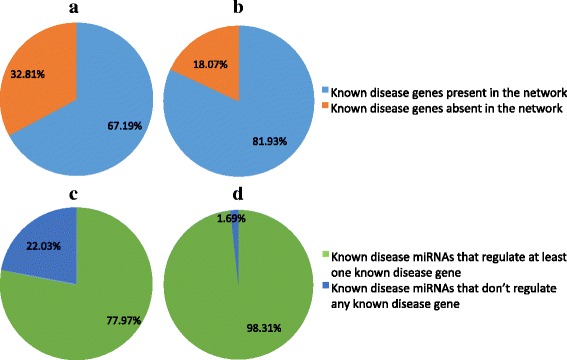
Heterogeneous miRNA networks contain known disease genes and known disease miRNAs, regulating known disease genes. **a** Percent of known disease genes in *HetermiRWalkNet-mutual.* **b** Percent of known disease genes in *HeterTargetScanNet-mutual.* **c** Percent of known disease miRNAs regulating disease genes in *HetermiRWalkNet-mutual.* **d** Percent of known disease miRNAs regulating disease genes in *HeterTargetScanNet-mutual.* Known disease genes and known disease miRNAs were collected from the OMIM [47] and miR2Disease [45] databases, respectively

Interestingly, the performance of RWRMTN on *HetermiRWalkNet-mutual* and *HeterTargetScanNet-mutual* is consistently higher than that of RWRMDA on *HomomiRWalkNet* and *HomoTargetScanNet* (two sample t-Test, *p* = 1.24 × 10^−6^ and 7.59 × 10^−9^, respectively). Average AUC values of RWRMTN on *HetermiRWalkNet-mutual* and *HeterTargetScanNet-mutual* are 0.819 and 0.853. Average AUC values of RWRMDA on *HomomiRWalkNet* and *HomoTargetScanNet* are 0.776 and 0.830. These results suggest that using mutual biological relations between miRNAs and their target genes helps improving the disease miRNA prediction. In other words, information contained in these biological relations is used less effectively when it is integrated as the degree of similarity between miRNAs in the homogeneous miRNA networks. In addition, the “disease module” idea can be expected to be more explicitly present in the heterogeneous miRNA networks. This principle is generally accepted for both miRNAs (functionally related miRNAs associate with phenotypically similar diseases [26, 27]) and genes (functionally related genes associate with phenotypically similar diseases [52–54]). Two miRNAs in a heterogeneous miRNA network are functionally related if they regulate the same target genes, but conversely, we can assume that two genes regulated by the same miRNAs can be functionally related too. To illustrate this, we investigated how many known disease genes are present as targets of miRNAs in our heterogeneous miRNA networks. We downloaded disease-gene associations from OMIM at the NCBI website [55] and retrieved 4388 associations between 3.284 disease phenotypes and 2,761 disease genes. Figure 4a and b shows that from these disease genes, 1,855 (~67.19%) and 2,262 (~81.93%) known disease genes are found as target genes in the heterogeneous miRNA networks respectively built from miRWalk and TargetScan. This implies that a large amount of disease genes are regulated by miRNAs. In addition, we investigated how many known disease miRNAs regulate known disease genes in the heterogeneous miRNA networks. Figure 4c and d shows that 92 (~77.97%) and 116 (~98.31%) out of 118 known disease miRNAs (see Materials and Methods) regulate at least one known disease gene in the heterogeneous miRNA networks constructed from the miRWalk and TargetScan databases. This indicates that a large amount of disease miRNAs regulate disease genes. The smaller fraction of known disease miRNAs found in *HetermiRWalkNet-mutual* compared to that in *HeterTargetScanNet-mutual* also indicates that disease miRNAs/genes in the former is less modularized compared to those in the later. Taken together, these results imply that disease-associated miRNAs and genes are located closely to each other in the heterogeneous networks. Therefore, considering them together by using heterogeneous miRNA networks when predicting novel disease-associated miRNAs can be advantageous.

#### Comparison between RWRMTN and RLSMDA

**Fig. 5:**
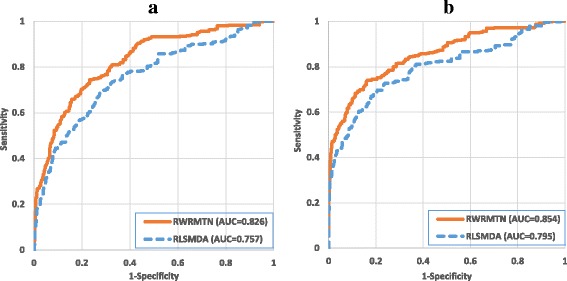
Comparison between RWRMTN and RLSMDA. The set of disease phenotypes and their associated miRNAs were collected from the miR2Disease database [45]. **a** MiRNA networks were constructed using the miRWalk database. **b** MiRNA networks were constructed using TargetScan database. Weight parameter α and restart probability γ were set to the optimal settings (α = 0.9 and γ = 0.7) for RWRMTN. For RLSMDA, we used the parameter settings (*η*_*M*_ = *η*_*D*_ = 1 and *w* = 0.9) reported in the study [33]

In addition to comparing with a representative network-based method, we also compared our method with RLSMDA [33], a state-of-the-art machine learning-based technique. To this end, we used the optimal set of parameters (α = 0.9 and γ = 0.7) for RWRMTN as obtained in the previous experiment. For RLSMDA, we used the parameter settings (*η*_*M*_ = *η*_*D*_ = 1 and *w* = 0.9) reported in the corresponding study [33]. Again, we used the ROC and AUC to compare these two methods on different databases of miRNA-target interactions. Figure 5 illustrates that RWRMTN (average AUCs are 0.826 and 0.854 in *HetermiRWalkNet* and *HeterTargetScanNet* respectively) outperforms RLSMDA (average AUCs are 0.757 and 0.795 in *HomomiRWalkNet* and *HomoTargetScanNet* respectively), suggesting that the explicit use of gene-miRNA interactions has an added value when predicting novel disease-related miRNAs. Comparing RWRMDA with RLSMDA, we used the best settings for RWRMDA and found the average AUCs of RWRMDA to be 0.789 (γ = 0.9) and 0.832 (γ = 0.3) in *HomomiRWalkNet* and *HomoTargetScanNet* respectively. This indicates that using functional miRNA interactions in RWRMDA results in inferior predictions compared to using miRNA-gene interactions in RWRMTN, but these predictions still outperform RLSMDA where no explicit network information is used.

#### Comparison between RWRMTN and RWRMDA, RLSMDA using 10-fold cross-validation

In previous section, we compare the performance of RWRMTN with that of RWRMDA and RLSMDA using leave-one-out cross validation (LOOCV). Considering that LOOCV is equivalent to n-fold cross validation (where n is number of known miRNAs of a given disease), this evaluation method is flexible and can be used to assess the prediction performance for any disease, even for those with only two known associated miRNAs. To show the robustness and stability of our method, we further test it with 10-fold cross validation on the TargetScan database. Due to this re-sampling method, only diseases known to be associated with at least 10 miRNAs can be taken into account. Using this criterion, only eight diseases in miR2Disease [45] were found to be eligible for validation. Additional file 2: Figure S1 shows the performance of the three methods using their respective optimal parameter settings (i.e., α = 0.9 and γ = 0.7 for RWRMTN, γ = 0.7 for RWRMDA, and *η*_*M*_ = *η*_*D*_ = 1, *w* = 0.9 for RLSMDA). It is obvious that RWRMTN (AUC = 0.840) outperforms both RWRMDA (AUC = 0.792) and RLSMDA (AUC = 0.753). We additionally used a larger disease-miRNA association database HMDD (version 2.0 [42]), containing 57 diseases eligible for performance assessment using 10-fold cross validation. Additional file 2: Figure S2 indicates that, again with optimal parameter settings for each method, the performance of RWRMTN (AUC = 0.896) is better than that observed for both RWRMDA (AUC = 0.875) and RLSMDA (AUC = 0.749).

### Identification of novel disease-associated miRNAs

To illustrate the power of RWRMTN to identify novel disease-associated miRNAs, we next tried to predict newly reported disease miRNAs in the experimentally verified disease-miRNA association HMDD database (version 2.0 [42]). As input for this analysis, we used known disease miRNAs as reported in the miR2Disease database [45]. First, we selected 23 disease phenotypes that were available in both databases. Then, for each disease phenotype, we used known associated miRNAs (as reported in the miR2Disease database) and their target genes as seed nodes in the *HeterTargetScanNet-mutual* network. We used the optimal parameter settings identified in the previous experiments (α = 0.9 and γ = 0.7) and ran our method to rank all remaining miRNAs in the network. After ranking, we selected the 100 top-ranked candidate miRNAs for each disease phenotype and checked whether they were reported in HMDD. Table 1 shows the results of this analysis. In total, 76 distinct novel disease miRNAs were predicted for the 23 disease phenotypes. We further tested per disease whether the selected 100 miRNAs were significant enriched for miRNAs reported in HMDD using a hypergeometric test [56]. For 18 out of the 23 disease phenotypes, we found the enrichment of the 100 predicted miRNAs for miRNAs reported in HMDD to be statistically significant (p ≤ 0.05). The remaining highly ranked miRNAs, for which no evidence about the association with the considered disease phenotypes yet exists, are candidates for further exploration in future studies (See in Additional file 1: Table S4). For several diseases, no significant enrichment of disease related miRNAs could be found. As Table 1 illustrates, this is due to the very small number of miRNAs that were associated with these diseases in the HMDD database. However, our top-ranked predictions contained miRNAs that have previously been associated with a disease, even though these associations were not present in HMDD. For example, hsa-miR-137 regulates the expression of the HTT gene, whose mutation leads to Huntington’s disease [57]. hsa-miR-15a and hsa-miR-27a are involved in human adipocyte differentiation and obesity [58]. Nicholas et al. [59] found that exposure to maternal obesity resulted in increased hepatic hsa-miR-29b. While investigating kidney tissue, which is known to be invoked in the etiology of essential hypertension, hsa-miR-181a and hsa-let-7c were found to be differentially expressed between kidneys of 15 untreated hypertensive and 7 normotensive white male subjects [60]. hsa-miR-181b and hsa-miR-181d were found to be differentially expressed between invasive and non-invasive non-functional pituitary adenoma [61]. Finally, hsa-let-7c-5p facilitated enterovirus 71 replication through viral subversion of cell signaling in rhabdomyosarcoma cells [62].

**Table 1:** MiRNAs present in the top-100 ranked candidate miRNAs that are known to be associated with diseases, as reported in the HMDD database. *P*-value is the result of the hypergeometric enrichment test

MIM ID	Disease	Overlap	Total in HMDD	*p*-value	Known disease miRNAs
150699	leiomyoma	2	3	0.012	hsa-miR-106b, hsa-miR-93
109800	bladder cancer	6	27	0.006	hsa-miR-17, hsa-miR-182, hsa-miR-200b, hsa-miR-200c, hsa-miR-20a, hsa-miR-27a
143100	huntington disease	1	7	0.376	hsa-miR-200c
601665	obesity	2	7	0.091	hsa-miR-17, hsa-miR-30e
145500	hypertension	3	15	0.069	hsa-let-7e, hsa-miR-17, hsa-miR-20a
600634	pituitary adenoma	1	14	0.065	hsa-miR-107
133239	esophageal cancer	8	51	0.017	hsa-let-7a, hsa-let-7b, hsa-let-7c, hsa-miR-19a, hsa-miR-200c, hsa-miR-203, hsa-miR-29c, hsa-miR-98
181500	schizophrenia	17	29	2.19×10^−13^	hsa-miR-106b, hsa-miR-137, hsa-miR-15a, hsa-miR-15b, hsa-miR-17, hsa-miR-181b, hsa-miR-195, hsa-miR-20b, hsa-miR-26b, hsa-miR-29a, hsa-miR-29b, hsa-miR-29c, hsa-miR-30a, hsa-miR-30b, hsa-miR-30d, hsa-miR-30e, hsa-miR-9
603956	cervical cancer	2	3	0.023	hsa-miR-20a, hsa-miR-424
155601	melanoma	34	130	8.97×10^−14^	hsa-let-7c, hsa-let-7d, hsa-let-7e, hsa-let-7f, hsa-let-7 g, hsa-let-7i, hsa-miR-106a, hsa-miR-106b, hsa-miR-137, hsa-miR-15a, hsa-miR-15b, hsa-miR-16, hsa-miR-17, hsa-miR-181a, hsa-miR-182, hsa-miR-195, hsa-miR-196a, hsa-miR-19a, hsa-miR-19b, hsa-miR-200b, hsa-miR-200c, hsa-miR-20a, hsa-miR-20b, hsa-miR-218, hsa-miR-23b, hsa-miR-27b, hsa-miR-30a, hsa-miR-30b, hsa-miR-30d, hsa-miR-30e, hsa-miR-429, hsa-miR-506, hsa-miR-9, hsa-miR-93
151400	leukemia	6	26	0.009	hsa-miR-17, hsa-miR-181a, hsa-miR-19a, hsa-miR-19b, hsa-miR-20a, hsa-miR-27a
268210	rhabdomyosarcoma	2	7	0.112	hsa-miR-106a, hsa-miR-29a
104300	alzheimer disease	10	16	9.36×10^−8^	hsa-miR-106b, hsa-miR-124, hsa-miR-125b, hsa-miR-128, hsa-miR-137, hsa-miR-17, hsa-miR-181c, hsa-miR-195, hsa-miR-20a, hsa-miR-9
256700	neuroblastoma	10	29	1.23×10^−5^	hsa-miR-106b, hsa-miR-124, hsa-miR-128, hsa-miR-19a, hsa-miR-19b, hsa-miR-20a, hsa-miR-27b, hsa-miR-340, hsa-miR-9, hsa-miR-93
113970	burkitt lymphoma	5	10	2.05×10^−4^	hsa-miR-17, hsa-miR-19a, hsa-miR-19b, hsa-miR-20a, hsa-miR-93
114500	colorectal cancer	26	120	3.04×10^−8^	hsa-let-7b, hsa-let-7c, hsa-let-7e, hsa-miR-106a, hsa-miR-137, hsa-miR-17, hsa-miR-181a, hsa-miR-181b, hsa-miR-182, hsa-miR-195, hsa-miR-19a, hsa-miR-19b, hsa-miR-200b, hsa-miR-200c, hsa-miR-20a, hsa-miR-218, hsa-miR-23a, hsa-miR-26a, hsa-miR-26b, hsa-miR-27b, hsa-miR-29a, hsa-miR-340, hsa-miR-497, hsa-miR-9, hsa-miR-93, hsa-miR-96
260350	pancreatic cancer	23	89	6.42×10^−9^	hsa-let-7b, hsa-let-7c, hsa-let-7d, hsa-let-7e, hsa-let-7f, hsa-let-7 g, hsa-let-7i, hsa-miR-106a, hsa-miR-128, hsa-miR-15a, hsa-miR-15b, hsa-miR-17, hsa-miR-181b, hsa-miR-182, hsa-miR-200a, hsa-miR-200c, hsa-miR-20a, hsa-miR-23a, hsa-miR-26a, hsa-miR-27a, hsa-miR-30c, hsa-miR-429, hsa-miR-96
211980	lung cancer	26	96	5.79×10^−9^	hsa-let-7i, hsa-miR-106a, hsa-miR-181a, hsa-miR-181b, hsa-miR-181c, hsa-miR-182, hsa-miR-19b, hsa-miR-200b, hsa-miR-200c, hsa-miR-206, hsa-miR-23a, hsa-miR-25, hsa-miR-27b, hsa-miR-301a, hsa-miR-30a, hsa-miR-30b, hsa-miR-30c, hsa-miR-30d, hsa-miR-30e, hsa-miR-32, hsa-miR-497, hsa-miR-9, hsa-miR-92a, hsa-miR-93, hsa-miR-96, hsa-miR-98
168600	parkinson disease	7	24	9.43×10^−4^	hsa-miR-19b, hsa-miR-29a, hsa-miR-29b, hsa-miR-29c, hsa-miR-30a, hsa-miR-30b, hsa-miR-30c
114480	breast cancer	41	170	1.96×10^−13^	hsa-let-7b, hsa-let-7c, hsa-let-7d, hsa-let-7e, hsa-let-7f, hsa-let-7 g, hsa-let-7i, hsa-miR-1, hsa-miR-106b, hsa-miR-137, hsa-miR-15a, hsa-miR-16, hsa-miR-181a, hsa-miR-181b, hsa-miR-182, hsa-miR-195, hsa-miR-19a, hsa-miR-19b, hsa-miR-202, hsa-miR-20b, hsa-miR-23a, hsa-miR-23b, hsa-miR-27b, hsa-miR-29a, hsa-miR-29b, hsa-miR-29c, hsa-miR-302a, hsa-miR-302b, hsa-miR-302c, hsa-miR-302d, hsa-miR-30a, hsa-miR-30b, hsa-miR-30c, hsa-miR-30d, hsa-miR-340, hsa-miR-497, hsa-miR-519d, hsa-miR-520b, hsa-miR-9, hsa-miR-93, hsa-miR-96
236000	lymphoma	14	47	5.64×10^−7^	hsa-miR-124, hsa-miR-133b, hsa-miR-15a, hsa-miR-17, hsa-miR-181a, hsa-miR-19a, hsa-miR-19b, hsa-miR-200b, hsa-miR-200c, hsa-miR-20a, hsa-miR-20b, hsa-miR-218, hsa-miR-26a, hsa-miR-29c
155255	medulloblastoma	9	57	0.013	hsa-miR-106a, hsa-miR-17, hsa-miR-181b, hsa-miR-182, hsa-miR-19a, hsa-miR-19b, hsa-miR-20a, hsa-miR-30a, hsa-miR-96
137215	gastric cancer	27	123	3.56×10^−9^	hsa-let-7f, hsa-let-7 g, hsa-miR-106a, hsa-miR-107, hsa-miR-124, hsa-miR-130a, hsa-miR-17, hsa-miR-181a, hsa-miR-181b, hsa-miR-182, hsa-miR-195, hsa-miR-200b, hsa-miR-200c, hsa-miR-20a, hsa-miR-27a, hsa-miR-27b, hsa-miR-29a, hsa-miR-30b, hsa-miR-30c, hsa-miR-340, hsa-miR-372, hsa-miR-373, hsa-miR-429, hsa-miR-497, hsa-miR-503, hsa-miR-519a, hsa-miR-9

## Conclusions

MiRNAs are known to have a strong impact on biological processes and play a pathogenic role in human diseases [63]. Therefore, the identification of novel disease-associated miRNAs is an essential part of biomedical research studying the underlying mechanisms of human diseases. Here we proposed a novel approach using a random walk with restart-based algorithm applied on mutual heterogeneous miRNA networks (RWRMTN), where contrary to previous efforts, miRNA-target gene relations were considered as bidirectional interactions, and the network used as input explicitly incorporates miRNA-target interactions. Experimental results demonstrate that our method achieves higher performance than a state-of-the-art network-based method (RWRMDA) that uses homogeneous miRNA networks, only containing miRNA nodes. We motivated that the superior performance of the proposed method can be partially attributed to the improved exploitation of the “disease module” principle. This concept is explicitly present in the heterogeneous miRNA networks used as input for our analysis, and we showed that a large amount of disease-associated miRNAs and disease related genes mutually interact with each other. Additionally, our method outperformed RLSMDA [33], a state-of-the-art machine learning-based method, confirming the added value of using network information when predicting novel disease related miRNAs. MiRNA-target interaction data predicted by in silico prediction tools typically have a high rate of false positive and false negative results. Therefore, we applied our method to two databases containing respectively predicted and experimentally validated miRNAs-target interactions. We could show that our method can achieve stable and high performance for both experimentally validated and predicted interaction data. Finally, using RWRMTN, we identified 76 miRNAs which were reported to be associated with 23 disease phenotypes in HMDD, an recent disease-miRNA association database.

## Additional files

Additional file 1: Table S1. A heterogeneous miRNA network constructed from the miRWalk database. **Table S2. **A heterogeneous miRNA network constructed from the TargetScan database. **Table S3. **List of disease phenotypes and their known associated miRNAs, as collected from the miR2Disease database. **Table S4. **miRNAs in the sets of 100 top-ranked candidate miRNAs that are not known to be associated with diseases in the HMDD database. (XLSX 7368 kb)

Additional file 2: Figure S1. Performance comparison between RWRMTN, RWRMDA and RLSMDA on miR2Disease database using 10-fold cross validation. **Figure S2.** Performance comparison between RWRMTN, RWRMDA and RLSMDA on HMDD database using 10-fold cross validation. (DOCX 185 kb)
