# Supplementary material for: Random walks on mutual microRNA-target gene interaction network improve the prediction of disease-associated microRNAs
Source: BMC Bioinformatics. 2017 Nov 14;18:479. doi: 10.1186/s12859-017-1924-1 (PMC5686822; doi:10.1186/s12859-017-1924-1)
Supplement: Supplementary file 2 — Performance comparison between RWRMTN, RWRMDA and RLSMDA on miR2Disease database using 10-fold cross validation. Figure S2. Performance comparison between RWRMTN, RWRMDA and RLSMDA on HMDD database using 10-fold cross validation. (DOCX 185 kb) [file 12859_2017_1924_MOESM2_ESM.docx]

**Random walks on mutual microRNA-target gene interaction networks improve the prediction of disease-associated microRNAs**

Duc-Hau Le^1,2^ Lieven Verbeke^3^, Le Hoang Son^4^, Dinh-Toi Chu^5,6^, Van-Huy Pham^7,^*

^1^Vinmec Research Institute of Stem Cell and Gene Technology, 458 Minh Khai, Hai Ba Trung, Hanoi, Vietnam

^2^School of Computer Science and Engineering, Thuyloi University, 175 Tay Son, Dong Da, Hanoi, Vietnam

^3^Department of Information Technology, Ghent University - imec, Ghent, Belgium.

^4^VNU University of Science, Vietnam National University, Hanoi, Vietnam

^5^Faculty of Biology, Hanoi National University of Education, Hanoi, Vietnam

^6^Institute of Research and Development, Duy Tan University, 03 Quang Trung, Da Nang, Vietnam

^7^Faculty of Information Technology, Ton Duc Thang University, Ho Chi Minh City, Vietnam

**Supplementary Figures**

**Figure S1.** **Performance comparison between RWRMTN, RWRMDA and RLSMDA on miR2Disease database using 10-fold cross validation.**

The performance was an average of AUC values over a set of eight disease phenotypes collected from *miR2Disease* database of known disease-miRNA associations and networks constructed from *TargetScan* database. Best settings for each method (i.e., α = 0.9 and γ = 0.7 for RWRMTN, γ = 0.7 for RWRMDA, and $\eta_{M}=\eta_{D}=1$, $w=0.9$ for RLSMDA) were used.

**Figure S2. Performance comparison between RWRMTN and RWRMDA and RLSMDA on HMDD data using 10-fold cross validation.**

The performance was an average of AUC values over a set of 57 disease phenotypes collected from *HMDD* database of known disease-miRNA associations and networks constructed from *TargetScan* database. Best settings for each method (i.e., α = 0.9 and γ = 0.7 for RWRMTN, γ = 0.7 for RWRMDA, and $\eta_{M}=\eta_{D}=1$, $w=0.9$ for RLSMDA) were used.

.
